# Fluorometric trace methanol detection in ethanol and isopropanol in a water medium for application in alcoholic beverages and hand sanitizers†

**DOI:** 10.1039/d1ra05201b

**Published:** 2021-09-09

**Authors:** Snigdha Roy, Sanju Das, Ambarish Ray, Partha Pratim Parui

**Affiliations:** Department of Chemistry, Jadavpur University Kolkata 700032 India parthaparui@yahoo.com +91-33-24146223 +91-9433490492; Department of Chemistry, Maulana Azad College Kolkata 700013 India; Department of Chemistry, Barasat Govt. College Kolkata 700124 India r_ambarish@yahoo.co.in +91-9836650180

## Abstract

Detection of methanol (MeOH) in an ethanol (EtOH)/isopropanol (^*i*^PrOH) medium containing water is crucial to recognize MeOH poisoning in alcoholic beverages and hand sanitizers. Although chemical sensing methods are very sensitive and easy to perform, the chemical similarities between the alcohols make MeOH detection very challenging particularly in the presence of water. Herein, the fluorometric detection of a trace amount of MeOH in EtOH/^*i*^PrOH in the presence of water using alcohol coordinated Al(iii)-complexes of an aldehydic phenol ligand containing a dangling pyrazole unit is described. The presence of MeOH in the EtOH/^*i*^PrOH causes a change of the complex geometry from tetrahedral (Td) to octahedral (Oh) due to the replacement of the coordinated EtOH/^*i*^PrOH by MeOH molecules. The Td-complex exhibited fluorescence but the Oh-species did not, because of the intramolecular photo-induced electron transfer (PET). By interacting the Oh species with water, its one MeOH coordination is replaced by a water molecule followed by the proton transfer from the water to pyrazole-N which generates strong fluorescence by inhibiting the PET. In contrast, the water interaction dissociates the Td-complex to exhibit fluorescence quenching. The water induced reversal of the fluorescence response from the decrease to increase between the absence and presence of MeOH is utilized to detect MeOH in an EtOH/^*i*^PrOH medium containing water with a sensitivity of ∼0.03–0.06% (v/v). The presence of water effected the MeOH detection and allows the estimation of the MeOH contamination in alcoholic beverages and hand sanitizers containing large amounts of water.

## Abstract 2

Detection of MeOH in EtOH/^*i*^PrOH and commercial samples based on water induced reversal of fluorescence response from the decrease to increase between the absence and presence of MeOH.

## Graphical abstract

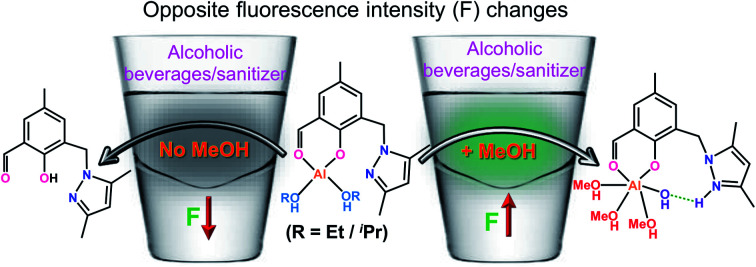

## Introduction

Worldwide, hundreds of economically constrained people are dying every year because of consumption of methanol (MeOH) contaminated illicit liquors.^1–3^ In the countryside, use of crude fermentation methods and improper distillation are the main culprits for the MeOH contamination in ethanol (EtOH). In some cases, unavoidable MeOH formation during standard fermentation processes is also a major concern.^4^ Consumption of MeOH beyond a certain permissible limit (1−2 mL per kg body mass) directly affects the central nervous system, by inhibiting the activity of cytochrome c oxidase, causing hypoxia, acidosis or even a painful death.^5–9^ Even a minute amount of MeOH ingestion, approximately 10 mL of dietary intake, is potent enough to cause some adverse effects.^10,11^ The use of much less expensive MeOH is a very common illegal practice used to alter the EtOH strength in alcoholic beverages to give a higher profit. Nevertheless, in recent times during the COVID-19 pandemic, a large number of poisonous MeOH containing hand sanitizers were seized worldwide, even after repeated warnings from the FDA.^12^ Because the use of costly EtOH and isopropanol (^*i*^PrOH) based hand sanitizers has significantly increased to help combat the COVID-19 pandemic, indiscriminate commercial production inevitably increases the chance of using MeOH containing cheaper hand sanitizers.^12^

The MeOH, EtOH and ^*i*^PrOH are all chemically similar in nature.^13–15^ Thus, using a reaction based chemical sensor, MeOH detection in commercial alcoholic beverages and hand sanitizers containing a large amount of EtOH/^*i*^PrOH as well as water is an extremely challenging task.^16–18^ In the search for an alternative method of detection, researchers focused on various other analytical procedures, such as different types of mass spectrometry (MS),^19–21^ gas chromatography,^22–24^ cyclic voltammetry,^25^ capillary electrophoresis,^26^ quartz crystal microbalances (QCMs) and so on.^27^ However, costly sophisticated instrumentation, the requirement of skilled technicians or tedious standardizations for the previous methods are major disadvantages for using them in routine analysis. In view of their cost-effectiveness and easy detection protocol, the reaction based chemical sensing methods are far superior detection techniques.

Fluorometric chemical sensing because of its ultra-high sensitivity is considered to be one of the most effective methods. Despite this, few organic fluorescent probes for MeOH are reported in the literature and those that are have certain limitations.^17,28–30^ Different materials have also been used as MeOH fluorosensors such as a supramolecular ionic material by Zhang *et al.*,^31^ a bimetallic lanthanide-organic framework by Du and co-workers,^32^ and nitrogen-doped oxidized carbon dots by Latha *et al.*^33^ In most of the cases MeOH is differentiated only from EtOH but not from ^*i*^PrOH. The detection is based on either an increase or decrease of the relative intensity changes between MeOH and EtOH but never in the opposite direction, that is an increase for one and a decrease for the other. In addition, the effect of a large amount of water in the sample being analyzed for MeOH, although useful in the preparation of alcoholic beverages and hand sanitizers, has not been thoroughly investigated. Thus, it is proposed that the MeOH detection based on water induced a reverse fluorescence response for the probe such as an increase in intensity in the presence of MeOH but a decrease in intensity in its absence for a EtOH/^*i*^PrOH medium.

The aldehydic phenol ligand (PPY) and its alcohol coordinated Al(iii)-complexes were strategically synthesized, and they exhibited a water mediated MeOH selective fluorometric response. The presence of MeOH in EtOH/^*i*^PrOH induces a change in the complex geometry from a fluorescent tetrahedral (Td) form to a weakly fluorescent octahedral (Oh) form, which is due to the exchange of coordinated EtOH/^*i*^PrOH by MeOH. The interaction of water with the Oh-species exhibited a strong fluorescence intensity because of the exchange of its one coordinated MeOH with a water molecule followed by an intramolecular proton transfer from the coordinated water to the ligand moiety. However, the less stable Td-complex in the absence of MeOH is dissociated by the water interaction to exhibit an intensity decrease. Such water induced opposite intensity changes between the absence and presence of MeOH are utilized to detect MeOH in EtOH/^*i*^PrOH and in alcoholic beverages/hand sanitizers in a water medium.

## Experimental

The general experimental procedures and materials are described on page S2 of the ESI.†

### Synthesis of PPY

Firstly, 3,5-dimethylpyrazole (1) and 2-hydroxy-3-(hydroxymethyl)-5-methylbenzaldehyde (2) were synthesized according to published procedures.^34,35^ To synthesize 3-(chloromethyl)-2-hydroxy-5-methylbenzaldehyde (3), 2 (0.5 mol) was taken in 2 mL of dichloromethane (DCM, CH_2_Cl_2_) and the suspension obtained was stirred. Freshly distilled SOCl_2_ in DCM was added drop wise (final ratio of SOCl_2_ : DCM = 1 : 1) under constant stirring. The yellow colored solution obtained was then stirred for another hour. Then the unreacted SOCl_2_ was removed. The solid residue was dissolved in 1 mL of DCM and the solution was further diluted in 1 mL of hexane. The diluted solution was then kept until it had evaporated to dryness, which produced white colored crystals. Next, 1.84 g (10 mmol) of 3 was dissolved in 5 mL of dry THF. Then, 0.96 g (10 mmol) of 1 was taken in 20 mM of TEA. The solution of 1 was added drop wise into the solution of 3, and the mixture was stirred for 24 h. The solution was extracted with brine solution and activated by Na_2_SO_4_ to obtain the desired product (PPY), which was further purified using column chromatography. ^1^H-NMR (DMSO-*d*_6_, 400 MHz): 2.08 (s, 3H, ArCH̲_3_), 2.23 (s, 6H, Py-2CH̲_3_), 2.51 (solvent residual peak), 3.33 (due to trace H_2_O), 5.14 (s, 2H, CH̲_2_-Ar), 5.86 (s,1H, Py-C=CH̲), 6.98 (s,1H, ArH̲), 7.49 (s,1H, ArH̲), 10.08 (s, 1H, CH̲=O), 11.12 (s,1H, ArOH̲) ppm. ^13^C-NMR (DMSO-*d*_6_, 75 MHz): 10.96, 13.82, 20.51, 39.51–40.90 (solvent residual peak), 46.51, 105.44, 121.82, 126.43, 129.27, 131.85, 136.95, 139.96, 139.79, 146.99, 156.36, 196.19 (Fig. S1 and S2, ESI†). ESI-MS^+^ for PPY in methanol: *m*/*z* calc. for [PPY + H]^+^: 245.281, found: 245.221 (Fig. S3A, ESI†).

### Generation of PPY/Al^3+^*in situ* complex and its reaction with water

For preparation of a stock solution of AlCl_3_ (1 mM), appropriate amounts of anhydrous AlCl_3_ were taken in different alcohol mediums and the mixture was vortexed until completely solubilized. Stock solutions of PPY (1 mM) in each alcohol medium were prepared separately. The alcohol medium was kept the same for the preparation of stock solutions and reaction medium. A portion (10 μL or 4 μL) of the stock solution of PPY (final concentration: 10 μM or 2 μM) was added to each reaction medium (final volume 2 mL) containing various amounts of AlCl_3_ (5–200 μM) in the absence or presence of water and/or MeOH in alcohol mediums under constant stirring, and the time-dependent PPY/Al^3+^ complex formation kinetics were monitored using UV-vis absorption and fluorescence studies at 25 °C. A diluted solution of PPY/Al^3+^ (0.1 μM PPY + 5 μM AlCl_3_) was used for the limit of detection (LOD) studies.

### UV-vis absorption and fluorescence studies

The UV-vis absorption and fluorescence studies were carried out in a double beam spectrophotometer (TCC-240A, Shimadzu, Japan) and spectrofluorometer (LS 55, PerkinElmer). The fluorescence spectra were obtained upon excitation at 402 nm (excitation band-pass: 10 or 8, and emission band pass: 2 or 8). Time-dependent fluorescence intensities at 505 nm were monitored for up to 60 min upon excitation at 405 nm while maintaining the same excitation and emission band-pass. The measuring solutions were filtered using a 0.45 mm filter (Millex, Millipore). The data reproducibility was checked using multiple measurements.

The LOD for MeOH was obtained as:^36^Detection limit (LOD) = 3*σ*/*k*,where *σ*, and *k* represent the experimental standard deviation and slope value of the linear fitting, respectively.

The fluorescence quantum yields were measured according to a procedure described earlier.^37^

### Theoretical calculations

For structural optimization, density function theory (DFT) calculations were performed with the Gaussian 09 Program.^38^ Time-dependent DFT (TD-DFT) calculations were performed to obtain UV-vis absorption parameters of different species. The structural optimizations were carried out by considering the B3LYP exchange-correlation functional and the 6-31G basis function.

## Results and discussion

### Probe design for MeOH detection

**Scheme 1:**
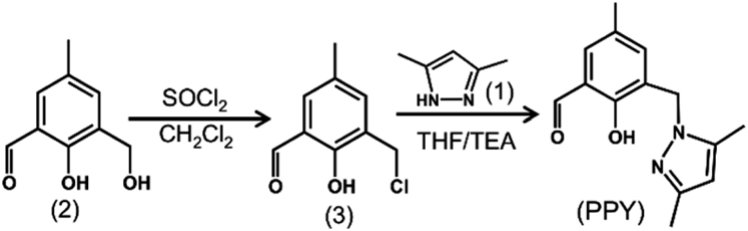
Synthesis route of PPY.

The synthesis route of the Al^3+^ binding aldehydic phenol ligand consisting of a dangling pyrazole unit (PPY) is shown in Scheme 1. It has recently been reported that the Al^3+^ ion exhibits a strong complex formation affinity with phenolic Schiff-base molecules by binding to phenolic-O and imine-N in alcohol solvents, and the rest of the Al(iii)-coordination sites were filled by alcohol molecules.^39^ In this research, the aldehydic moiety was deliberately not converted into the corresponding imine functionality of PPY, in order to achieve a reduced complex formation affinity due to the weaker interaction ability of aldehydic-O than that of the imine-N. Thus, upon the addition of a trace amount of the MeOH in the EtOH/^*i*^PrOH medium, a spontaneous conversion from a structurally fragile Td geometry to a relatively stable Oh symmetrical PPY/Al^3+^ complex is possible due to the exchange of coordinated EtOH/^*i*^PrOH by MeOH molecules. The reaction of water with PPY/Al^3+^ induces a fluorescence increase for the Oh species, but an intensity decrease for the Td complex. The MeOH induced the reversal of the fluorescence intensity change due to the change of the Al(iii) geometry which was utilized for the detection of trace MeOH in EtOH/^*i*^PrOH.

### The PPY/Al^3+^ complex formation and its interaction with water

**Fig. 1:**
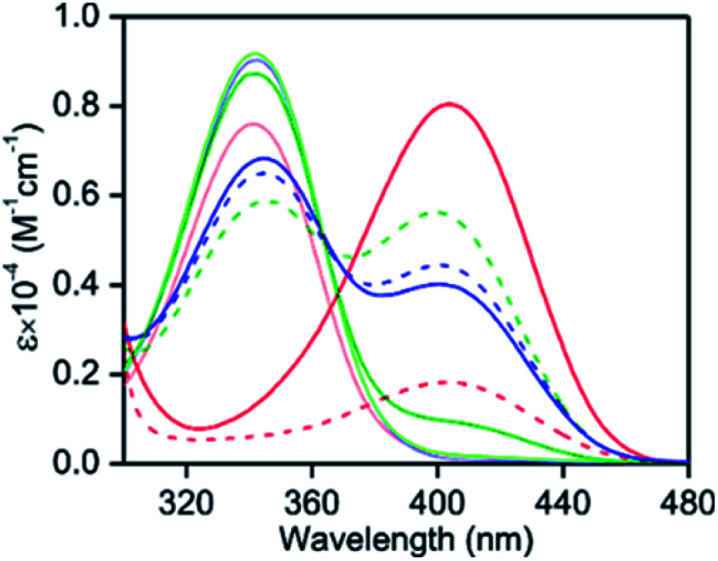
The UV-vis absorption spectra of PPY (10 μM) in the presence (solid lines) and absence (broken lines) of 2.5% (v/v) water containing anhydrous AlCl_3_ (200 μM) at 25 °C: red, MeOH; blue, EtOH and green, ^*i*^PrOH. The spectra were collected during the 60 min of AlCl_3_ addition. The spectra of PPY in the absence of AlCl_3_ and water are depicted in their respective light colors for comparison.

In the presence of anhydrous AlCl_3_ (200 μM), the UV-vis absorption intensity at ∼340 nm for PPY (10 μM) in alcohol solvents decreased gradually with time (up to 60 min), upon the formation of a new intensity at 405−410 nm through an isosbestic point at ∼378 nm (Fig. 1 and S4, ESI†), indicating that PPY was involved in a complex formation reaction with the Al^3+^ ion by a kinetically slow process. The amount of complex formation was evaluated directly by judging the relative intensity changeover from ∼340 nm to the 405–410 nm absorption band, because both intensities are not overlapped by each other (Fig. 1). However, to estimate the equilibrium between PPY and its Al(iii)-complex in the presence of various amount of AlCl_3_ (20–200 μM), the intensity values when the reaction attained equilibrium in nearly in 60 min were evaluated (Fig. 1 and S5, ESI†). It should be noted that a large amount of Al^3+^ (∼200 μM, 20 equiv.) was required to react all the PPY with Al^3+^ in an MeOH medium (Fig. 1 and S5, ESI†), which suggested that the interaction of PPY with the Al^3+^ ions was not only kinetically slow but also thermodynamically weak in nature. However, large fractions of unreacted PPY ∼60% in EtOH and ∼50% in ^*i*^PrOH medium were identified in the presence of the same concentration of Al^3+^ (20 equiv.) (Fig. 1). This result indicates that the complex formation affinity was reduced even more in the EtOH/^*i*^PrOH than in the MeOH medium.

In spite of inadequate complex formation in the EtOH or ^*i*^PrOH solvents, the intensity at ∼405 nm for the PPY/Al^3+^ complex was ∼3-fold larger, *i.e.*, there was a 6–7 times higher molar extinction coefficient (*ε*) value (∼1.1 × 10^4^ M^−1^ cm^−1^), than that observed in MeOH (∼0.17 × 10^4^ M^−1^ cm^−1^) (Fig. 1). Although phenolate-O and Al^3+^ bond formation was quite obvious, the formation of aldehydic-O with the Al^3+^ bond was assured by an up-field ^1^H-NMR chemical shift from ∼9.91 to 9.53 ppm, which was presumably due to an Al^3+^ binding induced, increased negative charge density at the aldehydic-O (Fig. S6 and S7 ESI,† compare with the theoretical calculation section). Furthermore, a 1 : 1 PPY to Al^3+^ binding with a reflection of coordinated alcohol molecules (maximum up to four MeOH molecules (*m*/*z* calc. for [PPY + 4MeOH + Al + Cl]^+^: 433.873, found: 433.912) and two EtOH molecules (*m*/*z* calc. for [PPY + 2EtOH + Al + Cl]^+^: 397.854, found: 397.823)) were recognized in the ESI-MS^+^ studies (Fig. S3B and D ESI†)). The results indicated that the saturation of the Al(iii)-coordination was effected by the solvent alcohol molecules. The reaction of MeOH (1–20% (v/v) with the solvent coordinated PPY/Al^3+^ in the presence of unreacted PPY in EtOH/^*i*^PrOH showed a gradual decrease of both UV-vis intensities at ∼340 nm of unreacted PPY and at ∼403 nm of the PPY/Al^3+^ complex due to newly formed MeOH coordinated complexes and a replacement of coordinated EtOH/^*i*^PrOH by MeOH molecules in the solvent coordinated PPY/Al^3+^, respectively, (Fig. S8, ESI†). The results justified the proposition that the stability or formation affinity was higher for MeOH coordinated PPY/Al^3+^ than for the EtOH/^*i*^PrOH coordinated one.

The interaction of the PPY/Al^3+^ complex with water molecules in EtOH/^*i*^PrOH medium showed an increase of absorption intensity at ∼340 nm whereas a decrease in intensity at ∼405 nm indicated the dissociation of the complex (Fig. S9, ESI†). However, a similar water interaction in the MeOH medium caused a large increase of absorption intensity (∼4-fold) at 405 nm without generating any absorption band at ∼340 nm for free PPY (Fig. 1). This result shows that water reacted with the Al(iii) center in the MeOH coordinated PPY/Al^3+^ complex without disturbing the PPY and Al(iii) interaction. Because of the greater stabilities of MeOH coordinated species, an incorporation of a water molecule in the Al(iii) coordination site may occur by it replacing one coordinated MeOH molecule, and this phenomenon was verified from the ESI-MS^+^ measurements (*m*/*z* calc. for [PPY + 3MeOH + H_2_O + Al + Cl]^+^: 419.842, found: 419.762) (Fig. S3C, ESI†).

### Solvent alcohol/water induced fluorescence response for PPY/Al^3+^

**Fig. 2:**
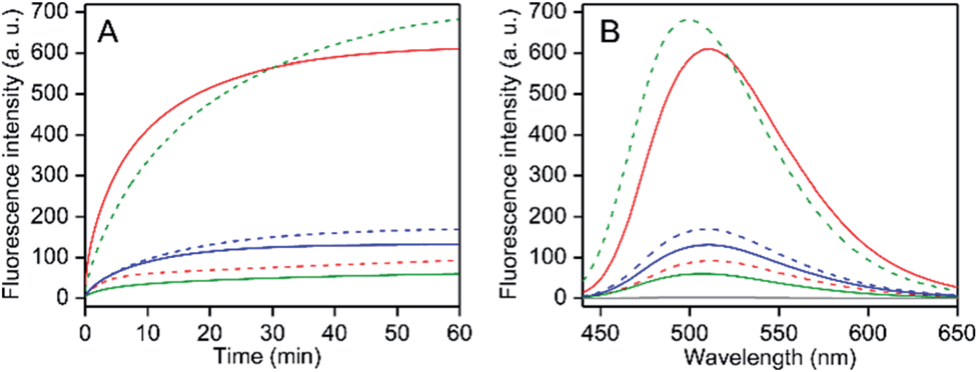
(A) Time-dependent fluorescence intensity changes at 505 nm upon the addition of anhydrous AlCl_3_ addition (50 μM), and (B) fluorescence spectra in 60 min of AlCl_3_ addition in various alcohol solvents in the presence (solid line) and absence (broken line) of 2.5% (v/v) water containing PPY (2 μM) at 25 °C: red, MeOH; blue, EtOH and green, ^*i*^PrOH. The spectrum in the absence of PPY is shown in grey (B). The excitation wavelengths were 405 nm in both (A and B).

**Fig. 3:**
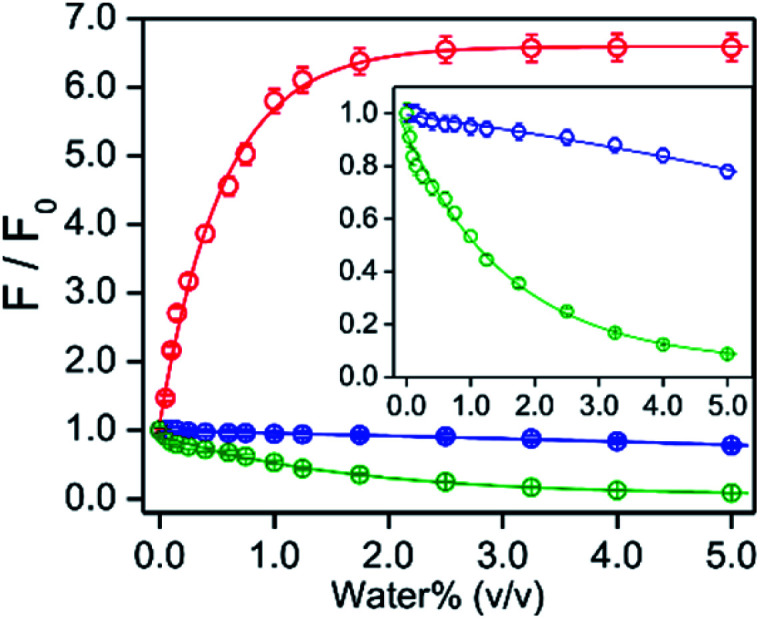
The ratio of fluorescence intensities at 505 nm for the PPY/Al^3+^ complex in the presence of various amounts of water% and its absence are plotted with the value of water% (v/v) in different alcohol mediums at 25 °C: red, MeOH; blue, EtOH and green, ^*i*^PrOH. The intensity values in the absence and presence of different water% are collected in 60 min of anhydrous AlCl_3_ (50 μM) addition in the medium containing PPY (2 μM). Inset: the *Y*-axis expanded plots for EtOH and ^*i*^PrOH medium are shown for clarity. Excitation and emission wavelength were 405 and 505 nm, respectively. The data points for each alcohol solvent are fitted using a single exponentially-fitted method. The average value for each data point is obtained from triplicate measurements (*n* = 3).

The PPY exhibited no fluorescence intensity. With an addition of AlCl_3_ (50 μM, 25 equiv.) in separate different alcohol mediums (MeOH, EtOH or ^*i*^PrOH) containing PPY (2 μM), the fluorescence intensity at ∼510 nm was enhanced gradually with time until the intensity was nearly saturated in ∼60 min of Al^3+^ addition (Fig. 2A). However, the saturated intensity value varied widely depending on the alcohol medium. Compared to MeOH, ∼8- and 2-fold larger intensities were detected in ^*i*^PrOH and EtOH, respectively, (*ϕ*_F_ ∼ 0.013 for MeOH, ∼0.025 for EtOH, and ∼0.102 for ^*i*^PrOH) (Fig. 2). Interestingly, the interaction of water with the PPY/Al^3+^ complex exhibited an increase of intensity in the MeOH medium but an intensity decrease in the EtOH/^*i*^PrOH medium (Fig. 2). An intensity increase of about 6-fold was observed in the MeOH medium containing ∼1.2% (v/v) water, whereas the intensity increased maximally up to ∼6.7-fold (*ϕ*_F_ ∼ 0.09) in the presence of ∼2.5% water (Fig. 3). In contrast, intensity quenching, almost completely in ^*i*^PrOH and ∼20% in EtOH solvents was detected by the addition of 5% water (Fig. 3). Similarly to the EtOH/^*i*^PrOH solvent, the water induced fluorescence decrease was noticed for other alcohols (*n*-PrOH, ^*t*^BuOH, *n*-hexanol) (Fig. S10, ESI†). Therefore, MeOH is a unique alcohol to use to show the water induced fluorescence increase.

### DFT theoretical calculations: complex structure *vs.* optical response

**Scheme 2:**
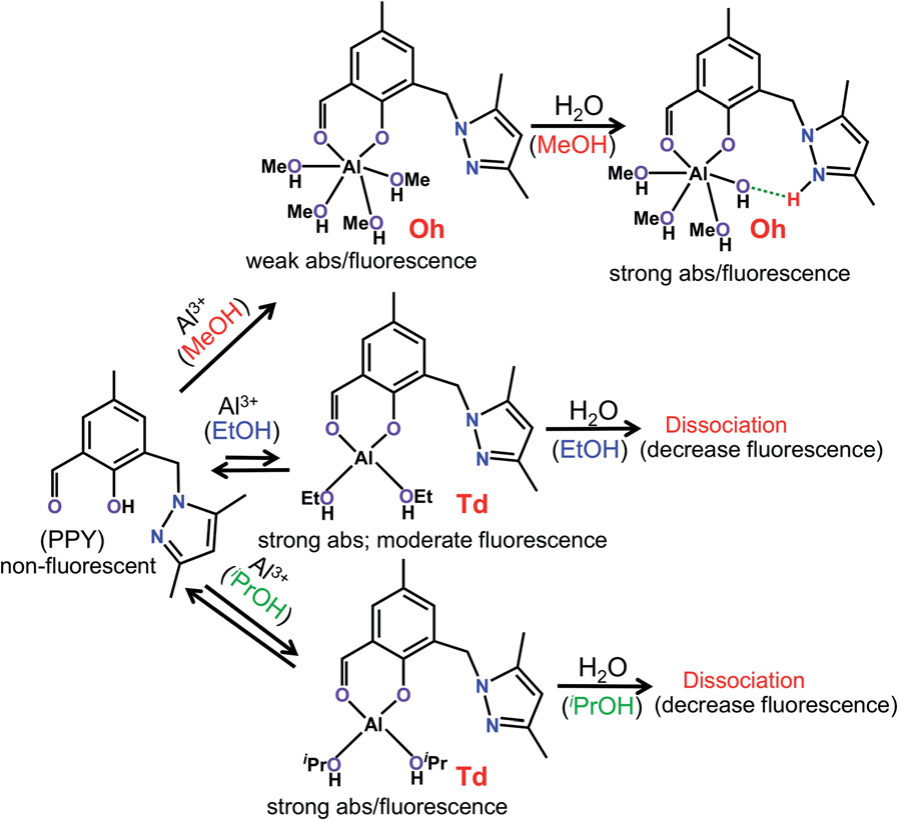
Mechanistic view of alcoholic solvent selective formation of geometrically different PPY/Al^3+^ complexes and their reaction with water molecules. The Al(iii) coordination saturation for the octahedral (Oh) geometry in MeOH and the tetrahedral (Td) geometry in EtOH/^*i*^PrOH medium are achieved by the coordination of four MeOH and two EtOH/^*i*^PrOH molecules, respectively. The coordination geometry dependent relative UV-vis absorption (abs), and the emission parameters of the PPY/Al^3+^ complex are shown.

The Al(iii) can exist as both Oh and Td geometric forms,^40–42^ where the Oh symmetry is more preferred than the Td symmetry.^41,42^ According to the results of the ESI-MS^+^ studies, coordination of four MeOH and two EtOH molecules in the respective solvents were identified (Fig. S3, ESI†). Because PPY was acting as 1 : 1 bi-dentate ligand for Al^3+^, the coordination of the four MeOH molecules was related to the Oh geometry of Al(iii). However, the same number of alcohol molecules binding for bigger EtOH or ^*i*^PrOH or any other alcohol molecules may not be a steric fit around the Al(iii) coordination sphere, thus a less stable Td structure which would allow two EtOH/^*i*^PrOH molecules was the most likely to occur (Scheme 2).

**Fig. 4:**
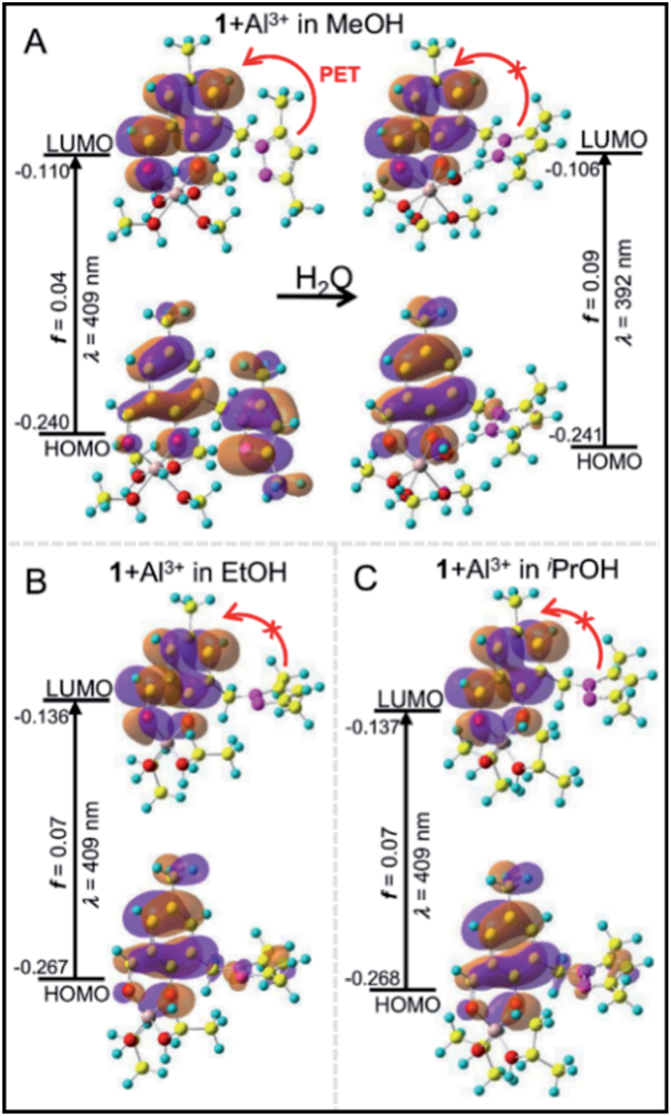
Frontier molecular orbital (FMO) profiles including different calculated UV-vis absorption parameters of MeOH (A: left upper panel) and MeOH/H_2_O (A: right upper panel) coordinated Oh. The EtOH (B: left lower panel) and ^*i*^PrOH (C: right lower panel) coordinated Td complexes based on DFT and TD-DFT (B3LYP/6-31G) calculations.

Using a DFT based theoretical calculation, it was identified that a possible Oh to Td structural interconversion for PPY/Al^3+^ was responsible for the alcoholic solvent dependent changes in UV-vis absorption and fluorescence properties, both in the presence and absence of water. The ground state geometries of four MeOH and two EtOH/^*i*^PrOH molecules coordinated Oh and Td complexes, respectively, with common phenolic-O and aldehydic-O coordination were optimized using B3LYP density function and a 6-31G basis set. The UV-vis absorption properties for the Oh and Td structures were evaluated using the TD-DFT calculations on the optimized ground state structures. The calculated HOMO to LUMO electronic transitions at ∼409 nm for both Oh and Td structures corresponded well with the respective experimental absorption wavelengths (Fig. 1, 4 and Scheme 2). In a similar way to the experimentally observed UV-vis intensity increase at ∼405 nm obtained by changing the solvent medium from MeOH to EtOH/^*i*^PrOH, the HOMO → LUMO oscillator strength (*f*_cal_) for the MeOH coordinated Oh geometry (∼0.04) was found to be significantly lower than that detected for the EtOH/^*i*^PrOH coordinated Td geometry (∼0.07) (Fig. 4). When one coordinated MeOH close to the pyrazole-N was replaced by a water molecule, the optimized structure showed a proton transfer reaction from the coordinated water molecule to pyrazole-N, and a large increase of *f*_cal_ from ∼0.04 to 0.09 was detected (Fig. 4 and Scheme 2). The increase of *f*_cal_ agreed well with the experimentally observed water induced large increase of UV-vis intensity in the MeOH medium (Fig. 1).

The efficient PET process from the pyrazole unit to the aldehydic phenol chromophore moiety made the PPY non-fluorescent (Fig. S11, ESI†). For the MeOH coordinated Oh structure, the PET process did not disturb it significantly, and thus a weak fluorescence intensity was observed experimentally (Fig. 2 and 4). However, the electron distribution in both HOMO and LUMO for EtOH or ^*i*^PrOH coordinated Td-species centered mostly at the aldehydic phenol chromophore, and the resultant suppression of the PET process made the Td complex highly fluorescent (Fig. 2, 4 and Scheme 2). Most interestingly, the calculations also identified that the PET process in the water substituted Oh species was eliminated, which clarified the probable reason for the water induced large increase of fluorescence intensity in the MeOH medium. All these studies suggested that the change of Al(iii) geometry from Oh to Td may be responsible for the alcohol solvent dependent change in optical response for the PPY/Al^3+^ complex.

### Detection of MeOH in EtOH or ^*i*^PrOH in the presence of water

It was found that the addition of water induced a fluorescence increase for MeOH coordinated Oh PPY/Al^3+^, whereas the intensity decreased for the EtOH/^*i*^PrOH coordinated Td complex (Fig. 3). An intensity increase of about 6.7-fold was found in MeOH/water mixed medium, which remained unaffected within a water% of ∼2.5%–11.0% (v/v), although the intensity decreased gradually as the water% was further increased (Fig. 3 and S12, ESI†). However, for the water% amount above ∼75%, the intensity value was found to be less in comparison to that observed in the absence of water (Fig. S12, ESI†). It was also observed that the coordinated solvent in the PPY/Al^3+^ complex were replaced by MeOH from the EtOH/^*i*^PrOH molecules with a subsequent change of complex geometry from Td to Oh by the addition of MeOH in EtOH/^*i*^PrOH (Fig. 2, Scheme 2, and Fig. S8, ESI†). Additionally, the residual unreacted PPY existed after the completion of a complex formation in EtOH/^*i*^PrOH medium reacted further with Al^3+^ to form MeOH coordinated PPY/Al^3+^ in the presence of MeOH (Fig. S8, ESI†). Moreover, the presence of 10% MeOH in the solution with various EtOH/^*i*^PrOH to water ratios showed that the presence of water effected different extents of intensity increase up to 70% water (Fig. S13, ESI†). All these results strongly suggest that the relative percentage of MeOH coordinated Oh complex with respect to the EtOH/^*i*^PrOH coordinated Td species should be much higher even in the presence of a low amount of MeOH in EtOH/^*i*^PrOH. The water effected fluorescence intensity increased in the presence of various MeOH amounts was investigated for its potential use in the analytical detection of MeOH in EtOH/^*i*^PrOH.

**Fig. 5:**
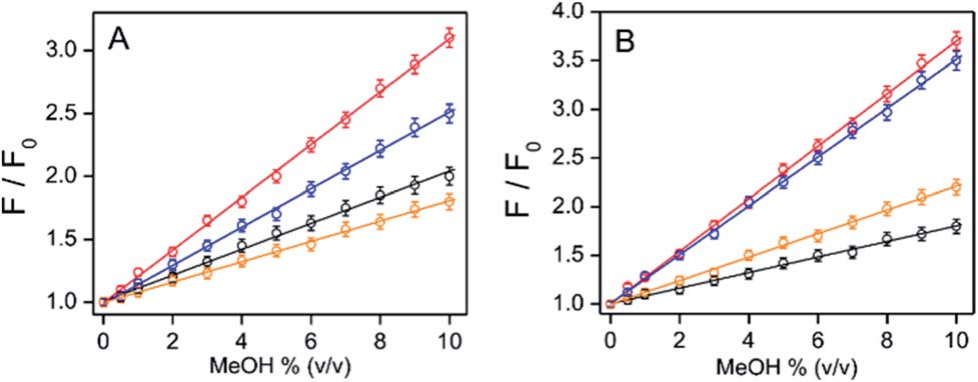
Relative fluorescence intensity changes between the presence and absence of MeOH for PPY/Al^3+^ are plotted with MeOH% (v/v) in (A) EtOH/water and (B) ^*i*^PrOH/water mixed medium containing various amounts of water% (v/v): black, 2.5%; red, 10%; blue, 25%; orange, 55%. The identical value of water% before and after the MeOH spike was maintained by an addition of an appropriate amount of water in the spiked sample. The intensity values in the absence and presence of various MeOH% were collected during the addition of AlCl_3_ (50 μM), over 60 min, to the medium containing PPY (2 μM). The data points for each solvent system are fitted with a linear equation. Excitation and emission wavelengths were 405 nm and 505 nm. The average value for each data point is obtained from triplicate measurements (*n* = 3).

In the EtOH/^*i*^PrOH medium containing water, the intensity ratios between the presence and absence of MeOH increased gradually with the increase of MeOH% (0.5–10% (v/v)) when the amount of any fixed water% value was within 2.5–55% (Fig. 5 and S14, ESI†). The relative intensity enhancements depended on the water%. For a solution containing 10% MeOH, the relative intensity increments were ∼2.0-, 3.1-, 2.5- and 1.5-fold for the EtOH system or ∼1.8-, 3.7-, 3.5- and 2.2-fold for the ^*i*^PrOH system in the presence of 2.5%, 10%, 25%, and 55% (v/v) of water, respectively (Fig. 5). The extent of the relative intensity increase with increasing MeOH% under various water% (2.5–55%) values followed a fairly good linear correlation (residual of fitting *χ*^2^ ∼ 0.99) for both the EtOH and ^*i*^PrOH systems, where the water% dependent slope values were estimated to be ∼0.10, 0.21, 0.15 and 0.08 for EtOH or ∼0.08, 0.26, 0.25 and 0.12 for ^*i*^PrOH in the presence of 2.5%, 10%, 25% and 55% water, respectively (Fig. 5). Using the linear calibration curve, the unknown amount of MeOH in the EtOH/^*i*^PrOH solvent containing various water% can be evaluated ratiometrically. It was evident that the water amount present in the solution played the most critical role for the MeOH detection sensitivity, in which the sensitivity was at maximum at a water amount of ∼10% (v/v) for both EtOH and ^*i*^PrOH. Notably, the MeOH (10% v/v) also induced an appreciable amount of increased fluorescence intensity for PPY/Al^3+^ and this was also observed in other alcohol mediums (*n*-PrOH, ^*t*^BuOH and *n*-hexanol) containing 5% water (Fig. S15, ESI†), which indicated that the MeOH detection selectivity of the PPY/Al^3+^ complex did not alter with the change of alcohol systems. However, to detect a low amount of MeOH or low LOD values, fluorescence studies were conducted in the presence of very low PPY/Al^3+^ concentrations (0.1 μM PPY and 4 μM Al^3+^) so that an appreciable fluorescence response can be observed even in the presence of much lower amount of MeOH. The fluorescence intensity changes in the presence of much lower amounts of MeOH (0.05–0.30%) are shown in Fig. S16 (ESI†). The LOD was evaluated using the equation: LOD = 3*σ*/*k* (see Experimental section). The LOD values for MeOH detection were estimated to be ∼0.03%–0.06% depending on the solvent compositions.

The water% dependency variation of the fluorescence response for MeOH was interpreted by combining the water% dependent various extent of intensity increase for MeOH medium in the absence of EtOH/^*i*^PrOH and the intensity decrease for EtOH/^*i*^PrOH in the absence of MeOH (Fig. 3 and S12, ESI†). The presence of a small amount of MeOH in the EtOH/^*i*^PrOH medium replaced coordinated EtOH/^*i*^PrOH with MeOH molecules in PPY/Al^3+^ to obtain a Td to Oh structural change. However, the existence of an EtOH/^*i*^PrOH coordinated Td complex and its water interaction induced intensity decrease cannot be neglected entirely in the interpretation of the fluorescence response values in the presence of various amounts of MeOH and water. The presence of a water induced ∼6.7-fold intensity increase remains unchanged between 2.5% and 10% of water for MeOH in the absence of EtOH/^*i*^PrOH (Fig. 3 and S12, ESI†) and the observed intensity was decreased by increasing the water% (2.5% to 10%) for EtOH/^*i*^PrOH in the absence of MeOH, which effects the enlargement of the MeOH detection slope value (∼0.10 to 0.21 for EtOH and ∼0.08 to 0.26 for ^*i*^PrOH) by the increase of water%. Significantly higher slope changes for ^*i*^PrOH medium: ∼3.7-fold compared to ∼2.1-fold for EtOH medium due to the increase of water% (2.5% to 10%) was rationalized by the increased water amount which induced a greater amount of intensity quenching for ^*i*^PrOH (∼90%) than the EtOH medium (45%) in the absence of MeOH (Fig. 5 and S12, ESI†). However, any further increase of water% from 10% to 55% produced a larger intensity decrease for MeOH in the absence of EtOH/^*i*^PrOH than for EtOH/^*i*^PrOH in the absence of MeOH (Fig. S12, ESI†), and thus a gradual decrease of the MeOH detection slope value from ∼0.21 to 0.08 for EtOH and ∼0.26 to 0.12 for ^*i*^PrOH was observed.

### The MeOH detection in alcoholic samples and sanitizers

The EtOH% in alcoholic beverages are dependent (5–70% (v/v)) on their classifications, and usually water is the rest of the liquid volume. However, according to WHO guidelines, the composition of hand sanitizers should be ∼80% EtOH (v/v) or 75% ^*i*^PrOH (v/v), glycerol (1.45% (v/v)), and H_2_O_2_ (0.125% (v/v)).^43^ Spiked MeOH% in high and low EtOH% containing vodkas (∼45% (v/v)) and wine (∼15% (v/v)) samples, respectively, were estimated. As the MeOH detection sensitivity at above 55% (v/v) of water was comparatively low (Fig. S12 and S13, ESI†), an external EtOH addition is required for the detection of MeOH in the wine samples. In addition, spiked MeOH was estimated both in the presence and absence of externally added EtOH to show the applicability of the method for alcoholic beverages containing higher EtOH%. However, the spiked MeOH amounts were estimated in EtOH- and ^*i*^PrOH-based hand sanitizers without any further addition of external EtOH/^*i*^PrOH.

**Fig. 6:**
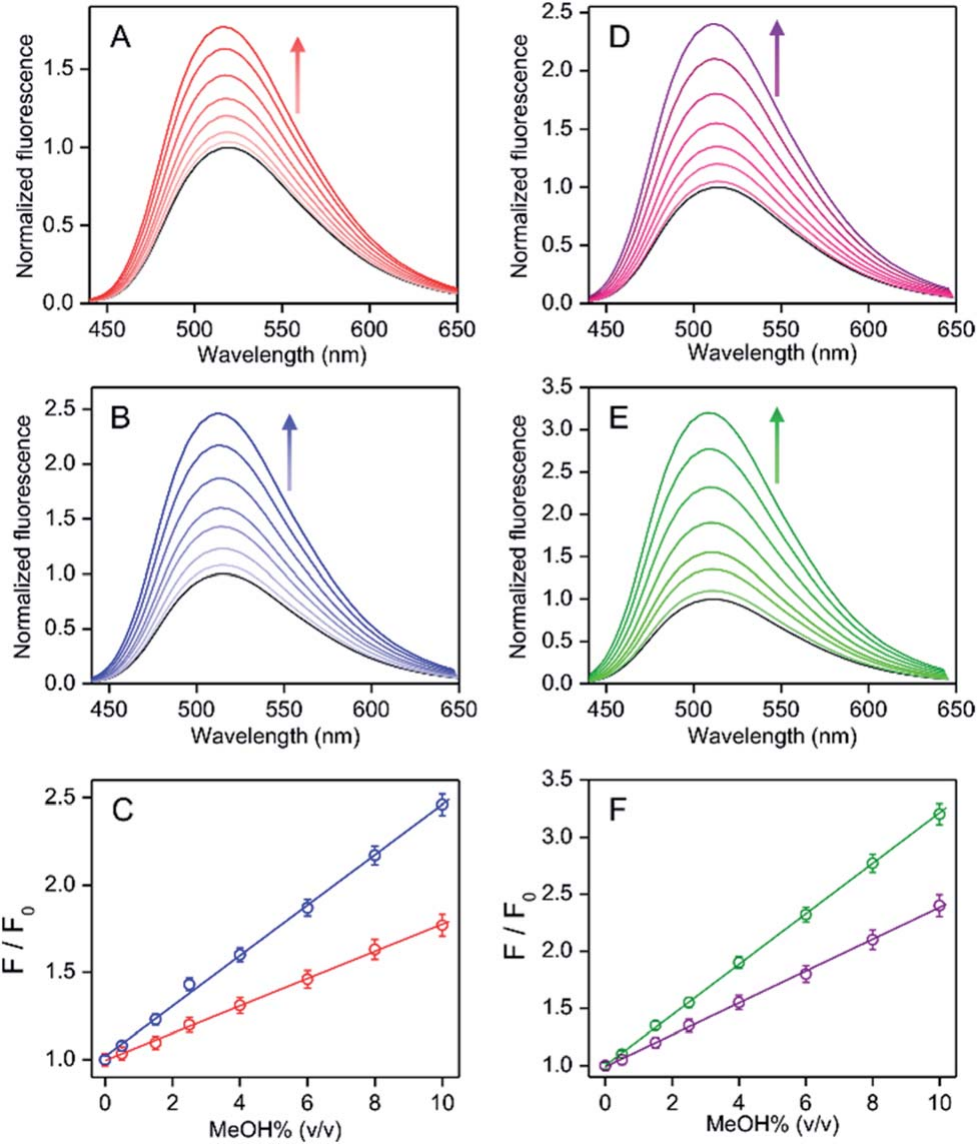
The relative fluorescence spectral changes between the presence and absence of MeOH for PPY/Al^3+^ with various MeOH spikes (0.5–10% (v/v)) in an alcoholic beverage (vodka: labelled EtOH% ∼ 45% (v/v)) in (A) the absence and (B) the presence of externally added 30% EtOH, and (D) EtOH or (E) ^*i*^PrOH-based hand sanitizers (labelled ^*i*^PrOH ∼75% and EtOH ∼80%) at 25 °C. The spectra in the absence of MeOH spikes are shown in black. The maximum intensity values for alcoholic beverages (C) and hand sanitizer (F) samples are plotted against the amount of the MeOH spikes. (A–C) Blue and red correspond to the presence and absence of externally added 30% EtOH, respectively. (D–F) Purple and green correspond to EtOH- and ^*i*^PrOH-based hand sanitizer, respectively. (A–F) The identical value of water% before and after of MeOH spike was maintained by using an appropriate amount of water addition in the spiked sample. The intensity increases with the increase of MeOH% are shown by arrows. The excitation wavelength was 405 nm. The average value for each data point is obtained from triplicate measurements (*n* = 3).

To observe the MeOH induced fluorescence intensity increase, the water% before and after MeOH spikes in the hand sanitizer samples were maintained by addition of an appropriate amount of water in the spiked MeOH sample. With the increase of MeOH spikes from 0.5% to 10% in the vodka sample in the presence and absence of externally added 30% EtOH (total water ∼25%), the relative fluorescence intensity between the presence and absence of MeOH was found to increase linearly from 1.04- to 1.77-fold and 1.08- to 2.45-fold, respectively (Fig. 6A–C). For a wine sample with the externally added 30% EtOH, the relative intensity also increased linearly from 1.05- to 1.78-fold (Fig. S17, ESI†), where the slope value of the linear plots ∼0.08 was found to be similar to that obtained for a known EtOH/water mixed medium (45% EtOH) or vodka (45% EtOH) sample (Fig. 5A). In addition, the slope values for vodka samples with 30% EtOH added externally (total EtOH, 75%) were also similar to the results obtained for the known 75% EtOH medium (Fig. 5A, 6B and C). All these results clearly showed that the presence of other chemicals in alcoholic beverages did not disturb the detection ability of the MeOH. Even without knowing the accurate water% value in the test sample, the estimation of MeOH contamination was possible from the correlation of fluorescence response of the test sample with the linear calibration plots for the corresponding MeOH free alcoholic beverages (Fig. 6C).

The fluorescence spectra for PPY/Al^3+^ in EtOH (80%) or ^*i*^PrOH (75%) and a water mixed medium remain unchanged by the addition of glycerol (1.45% (v/v)) or H_2_O_2_ (0.125% (v/v)), both in the presence and absence of MeOH (Fig. S18, ESI†), showing that the presence of glycerol and H_2_O_2_ in hand sanitizers did not affect the performance of the probe. The intensity increased linearly from ∼1.05 to 2.38 for EtOH-based sanitizer or from ∼1.08 to 3.22 for the ^*i*^PrOH-based sanitizer because of the increase of the amount of MeOH spiking from 0.5% to 10% under the identical water% condition. The observed slope value of ∼0.22 for the ^*i*^PrOH-based sanitizer and of ∼0.14 for the EtOH-based sanitizer were similar to that detected for the known 80% EtOH and 75% ^*i*^PrOH medium, respectively (Fig. 5 and 6D–F). Therefore, an unknown amount of MeOH contamination in hand sanitizers could be estimated by correlating the intensity value of the test sample with the known linear calibration line obtained for the EtOH (or ^*i*^PrOH) containing water or MeOH free standard for the EtOH (or ^*i*^PrOH)-based hand sanitizer.

As in the procedure described previously, a low level of MeOH contamination in alcoholic beverages and sanitizer could be estimated using a low probe concentration (0.1 μM PPY and 4 μM Al^3+^). The MeOH induced fluorescence spectral changes in the presence of a lower amount of MeOH spikes (0.06–0.18% for a vodka sample and 0.03–0.10% for the ^*i*^PrOH hand sanitizer) revealed that even a MeOH contamination of below 0.1 μM in alcoholic beverages and sanitizer can be estimated accurately by the present protocols (Fig. S19, ESI†). The efficiency of probe recovery was also verified by conducting EDTA induced fluorescence intensity quenching studies in vodka and EtOH-based hand sanitizers. For both samples, MeOH induced ∼90% of the increased intensity for PPY/Al^3+^ which was found to be quenched by the addition of EDTA, whereas the intensity recovered again upon further addition of Al^3+^ (Fig. S20, ESI†). The EDTA induced displacement of PPY from the PPY/Al^3+^ complex again participated in complexation with the freshly added Al^3+^ to regain the fluorescence intensity by the reaction with MeOH present in solution. Thus, the probe can be reused on several occasions.

## Conclusions

A sensitive fluorometric MeOH detection method was demonstrated in EtOH/^*i*^PrOH in a water medium using a 1 : 1 Al(iii)-complex of an aldehydic phenol ligand containing a pyrazole unit (PPY). The complex adopted the MeOH coordinated weakly fluorescent octahedral (Oh) geometry from the fluorescent tetrahedral (Td) structure by an addition of MeOH in the EtOH/^*i*^PrOH. The interaction of water with the Oh species causes a large fluorescence intensity increase due to the exchange of one coordinated MeOH by a water molecule, whereas a similar water interaction for the Td complex resulted in an intensity decrease due to its dissociation. The water mediated fluorescence intensity reversal due to the change in complex geometries by the addition of MeOH was utilized to detect MeOH in EtOH/^*i*^PrOH and various alcoholic beverages/hand sanitizers. Such water induced MeOH detection could be very useful industrially.

## Author contributions

Snigdha Roy: experimental, analysis and review and editing. Sanju Das: synthesis. Ambarish Ray: conceptualization, supervision, editing. Partha Pratim Parui: experimental, conceptualization, writing – original draft, supervision.

## Conflicts of interest

There are no conflicts of interest to declare.

## Supplementary Material

RA-011-D1RA05201B-s001

## References

[cit1] Jarwani S. B., Motiani P. D., Sachdev S. (2016). J. Emerg. Trauma Shock.

[cit2] Beauchamp G. A., Valento M. (2016). Emerg. Med. Pract..

[cit3] Paasma R., Hovda K. E., Tikkerberi A., Jacobsen D. (2007). Clin. Toxicol..

[cit4] Bindler F., Voges E., laugel P. (1988). Food Addit. Contam..

[cit5] Hantson P. E. (2006). Bull Mem Acad R Med Belg.

[cit6] Barceloux D. G., Bond G. R., Krenzelok E. P., Cooper H., Vale J. A. (2002). J. Toxicol. Clin. Toxicol..

[cit7] Liu J. J., Daya M. R., Mann N. C. (1999). J. Toxicol., Clin. Toxicol..

[cit8] Onder F., Ilker S., Kansu T., Tatar T., Kural G. (1998). Int. Ophthalmol..

[cit9] Kruse J. A. (2012). Crit. Care Clin..

[cit10] Moon C. S. (2017). Ann Occup Environ Med.

[cit11] Jacobsen D., McMartin K. E. (1986). Med. Toxicol..

[cit12] Welle L., Medoro A., Warrick B. (2021). Ann. Emerg. Med..

[cit13] Pereira P. F., Sousa R. M. F., Munoz R. A. A., Richter E. M. (2013). Fuel.

[cit14] Methanol: colorless, mp = −97.0 °C, bp = 64.7 °C, *d* = 0.79 g dm^3^, *n*_D_ (at 20 °C) = 1.33, dipole moment = 1.70 D, dielectric constant = 33, p*K*_a_ = 15.5

[cit15] Ethanol: colorless, mp = −114.3 °C, bp = 78.4 °C, *d* = 0.799 g dm^3^, *n*_D_ (at 20 °C) = 1.36, dipole moment = 1.69 D, dielectric constant = 24.6, p*K*_a_ = 15.9

[cit16] Mohr G. J., Lehmann F., Grummt U. W., Spichiger-Keller U. E. (1997). Anal. Chim. Acta.

[cit17] Lim S. H., Feng L., Kemling J. W., Musto C. J., Suslick K. S. S. (2009). Nat. Chem..

[cit18] Qin T., Liu B., Huang Y., Yang K., Zhu K., Luo Z., Pan C., Wang L. (2018). Sens. Actuators, B.

[cit19] Cordell L. R., Pandya H., Hubbard M., Turner A. M., Monks S. P. (2013). Anal. Bioanal. Chem..

[cit20] Allen T. M., Falconer T. M., Cisper M. E., Borgerding A. J., Wilkerson C. W. (2001). Anal. Chem..

[cit21] Shestivska V., Kolivoska V., Kubista J., Smith D., Spanel P. (2020). Rapid Commun. Mass Spectrom..

[cit22] Wang M. L., Choong Y. M., Su N. W., Lee M. H. (2003). J Food Drug Anal.

[cit23] Bursova M., Hlozek T., Cabala R. (2015). J. Anal. Toxicol..

[cit24] Broek J. V. D., Abegg S., Prastsinis S. E., Guntner A. T. (2019). Nat. Commun..

[cit25] Park D. S., Won M. S., Goyal R. N., Shim Y. B. (2012). Sens. Actuators, B.

[cit26] Santos M. S. F., Costa E. T. D., Gutz I. G. R., Garcia C. D. (2017). Anal. Chem..

[cit27] Wang C., Chen F., He X. W., Kang S. Z., You C. C., Liu Y. (2001). Analyst.

[cit28] Kumar V., Kumar A., Diwan U., Singh M. K., Upadhyay K. K. (2015). Org. Biomol. Chem..

[cit29] Wu Z., Fu X., Wang Y. (2017). Sens. Actuators, B.

[cit30] Zhao M., Yue Y., Liu C., Hui P., He S., Zhao L., Zheng X. (2018). Chem. Commun..

[cit31] Zhang L., Qi H., Wang Y., Yang L., Yu P., Mao L. (2014). Anal. Chem..

[cit32] Chen D. M., Sun C. X., Peng Y., Zhang N. N., Si H. H., Liu C. S., Du M. (2018). Sens. Actuators B.

[cit33] Latha M., Devi R. A., Bogireddy N. K. R., Rios S. E. S., Mochan W. L., Uribe J. C., Agarwal V. (2020). RSC Adv..

[cit34] Wiley R. H., Hexner P. E. (1951). Org. Synth..

[cit35] Gagne R. R., Spiro C. L., Smith T. J., Hamann C. A., Thies W. R., Schiemke A. K. (1981). J. Am. Chem. Soc..

[cit36] International Conference on Harmonization (ICH) of Technical Requirements for Registration of Pharmaceuticals for Human Use, Topic Q2 (R1): Validation of Analytical Procedures: Text and Methodology, 2005, http://www.ich.org

[cit37] Morris J. V., Mahaney M. A., Huber J. R. (1976). J. Phys. Chem..

[cit38] Frisch M. J. , *et al.*, Gaussian 09 Rev. A.1, Gaussian Inc., Wallingford CT, 2009

[cit39] Roy S., Das S., Majumder R., Ray A., Parui P. P. (2020). RSC Adv..

[cit40] Delpuech J.-J., Khaddar M. R., Peguy A. A., Rubini P. R. (1975). J. Am. Chem. Soc..

[cit41] Furia E., Beneduci A., Russo N., Marino T. (2018). New J. Chem..

[cit42] Malacaria L., Corrente G. A., Beneduci A., Furia E., Marino T., Mazzone G. (2021). Molecules.

[cit43] Guide to Local Production: WHO-recommended handrub for-mulations (PDF), World Health Organization, WHO/IER/PSP/2010.5, WHO April, 2010

