# Supplementary material for: Fluorometric trace methanol detection in ethanol and isopropanol in a water medium for application in alcoholic beverages and hand sanitizers
Source: RSC Adv. 2021 Sep 9;11(48):30093–101. doi: 10.1039/d1ra05201b (PMC9040838; doi:10.1039/d1ra05201b)
Supplement: RA-011-D1RA05201B-s001 [file RA-011-D1RA05201B-s001.pdf]

Electronic Supplementary Material (ESI) for RSC Advances.
This journal is © The Royal Society of Chemistry 2021

Supporting Information

Fluorometric trace methanol detection in ethanol and isopropanol in a water medium for application in alcoholic beverages and hand sanitizers

Snigdha Roy,[a] Sanju Das,[b] Ambarish Ray,*[,c] and Partha Pratim Parui*[,a]

Contents

1. General experimental procedures. …p S2
2. ^{1}H NMR spectra of PPY. …p S3
3. ^{13}C NMR spectra of PPY. …p S4
4. ESI-MS^{+} studies of PPY and PPY/Al^{3+} complex. …p S5
5. Time-dependent UV-vis spectra for PPY/Al^{3+} formation in MeOH. …p S6
6. UV-vis absorption spectra for PPY/Al^{3+} complex formation. …p S7
7. ^{1}H NMR spectra of PPY and PPY/Al^{3+} complex in MeOH. …p S8
8. Mulliken charge values for PPY and PPY/Al^{3+} complex. …p S9
9. MeOH induced complex formation in EtOH and ^{i}PrOH medium. …p S10
10. Water induced complex dissociation in EtOH and ^{i}PrOH mediums. …p S11
11. Effect of water on PPY/Al^{3+} complex formation abilities in alcohols. …p S12
12. Frontier molecular orbital (FMO) profiles of PPY. …p S13
13. Plots of complex formation amount *vs*. water% in different alcohols. …p S14
14. Effect of water% on MeOH induced fluorescence increase. …p S15
15. MeOH-spike induced fluorescence changes in EtOH and ^{i}PrOH medium. …p S16
16. MeOH detection selectivity studies in other alcohols. …p S17
17. MeOH induced fluorescence changes at low probe concentration. …p S18
18. MeOH% dependent fluorescence changes in wine sample. …p S19
19. Effect of glycerol and H_2O_2 on the fluorescence response of PPY/Al^{3+}. …p S20
20. Low amount of MeOH detection in commercial samples. …p S21
21. Probe recovery studies for the MeOH detection. …p S22

General Methods and instruments: Hydrazine hydrate, acetyl acetone, p-cresol, triethyl amine (TEA) and anhydrous $AlCl_3$ of analytical grade were procured from Sigma-Aldrich (USA). Analytical grade dichloromethane (DCM), tetrahydrofuran (THF), thionyl chloride ($SOCl_2$) solvents were purchased from E-Mark (India). Molecular sieves of 3Å pore size were obtained from Sigma-Aldrich (USA). HPLC grade alcohols (methanol (MeOH), ethanol (EtOH), isopropanol (^{i}PrOH), n-propanol (PrOH), tertiary-butanol, and *n*-hexanol) were also purchased from Sigma-Aldrich (USA), and the solvents were further dried over the 3Å molecular sieves. The solvents were stored under dry N_2 atmosphere in the presence of the molecular sieves. Throughout all experimental studies, Milli-Q water (Millipore) of conductivity 18.2 MΩ.cm was used.

^{1}H NMR studies of PPY in the absence and presence of anhydrous $AlCl_3$ were performed in DMSO-d_6 or CD_3OD with Bruker 400 MHz NMR spectrophotometer with considering tetramethylsilane (0 ppm) as an internal standard. A QTOF Micro YA263 mass spectrometer used to perform ESI-MS^{+} studies in the positive mode.

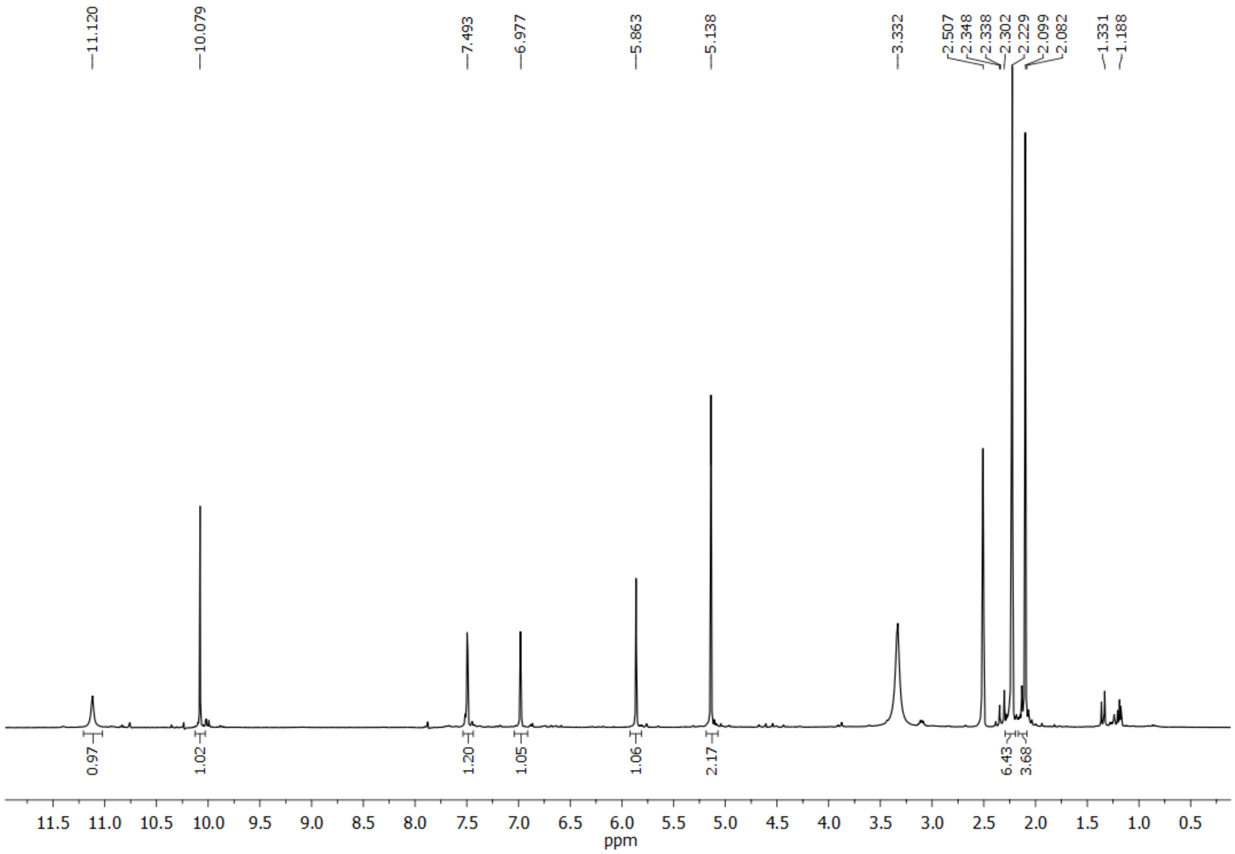

Fig. S1. ^{1}H-NMR spectrum of PPY in DMSO-d_6.

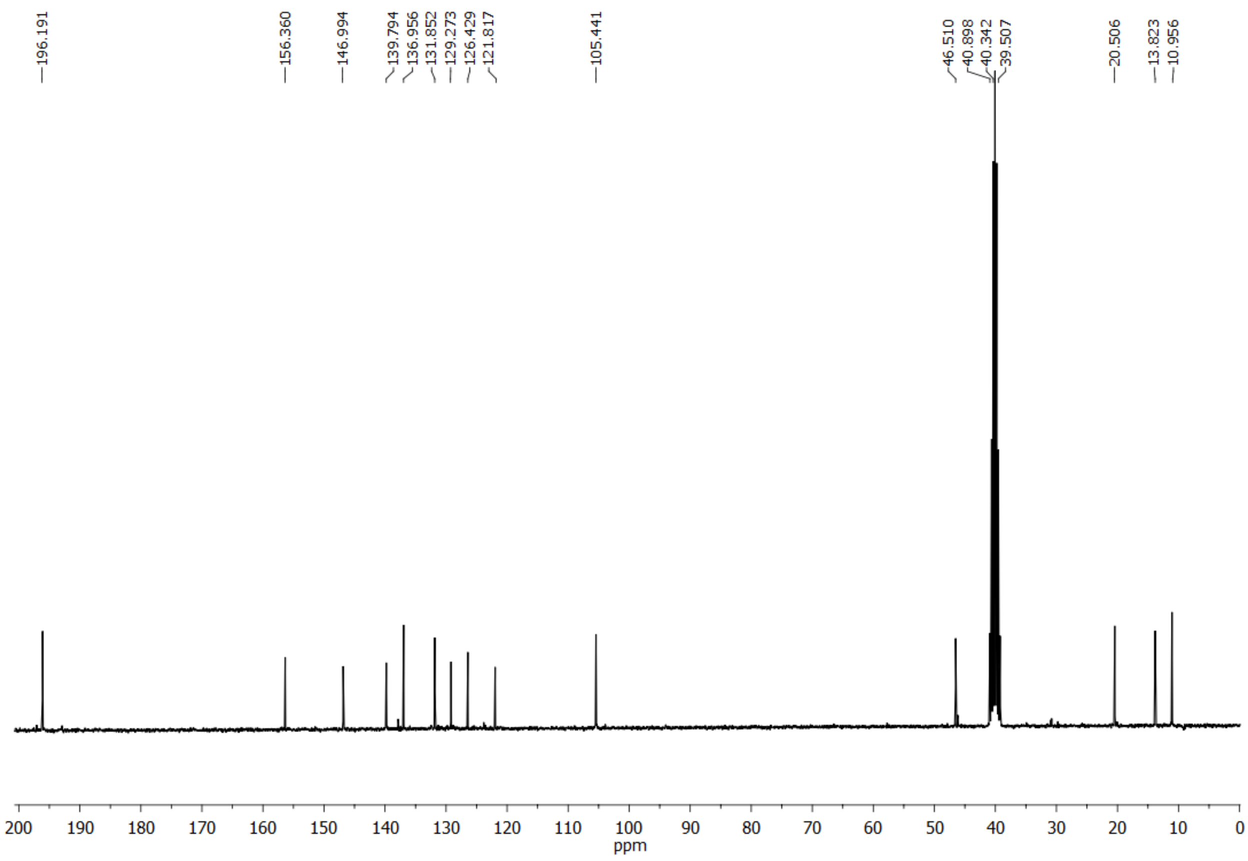

Fig. S2. ^{13}C-NMR spectrum of PPY in DMSO-d_6.

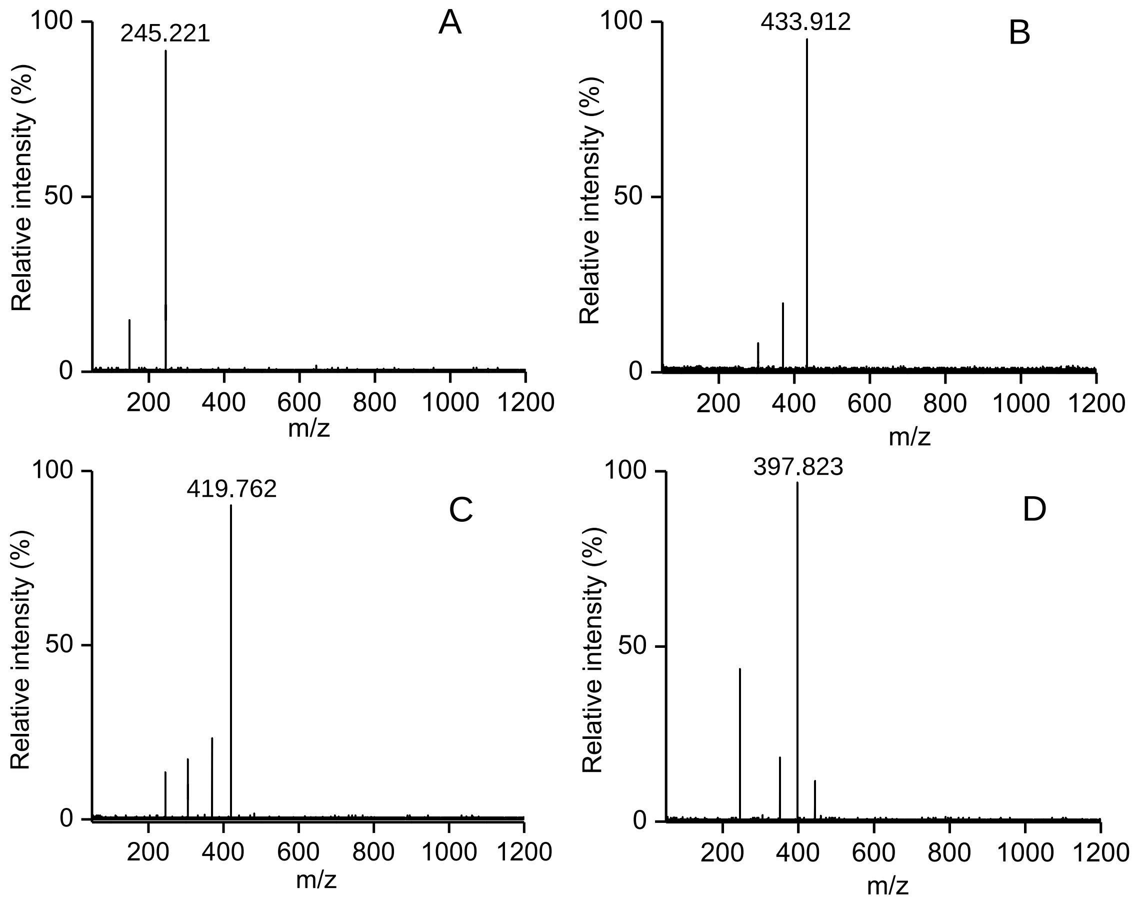

Fig. S3. ESI-MS$^+$ of PPY (A) (*m/z* for $[PPY+H]^+$: cal'd – 245.281 (obs'd – 245.221)) and PPY/Al^{3+} complex in (B) MeOH (*m/z* for $[PPY+Al+Cl+4CH_3OH]^+$: cal'd – 433.873 (obs'd – 433.912)), (C) MeOH/water (water: 2% (*v*/*v*)) (*m/z* for $[PPY+Al+Cl+H_2O+3CH_3OH]^+$: cal'd – 419.842 (obs'd – 419.762)) and (D) EtOH (*m/z* for $[PPY+Al+Cl+2C_2H_5OH]^+$: cal'd – 397.854 (obs'd – 397.823)).

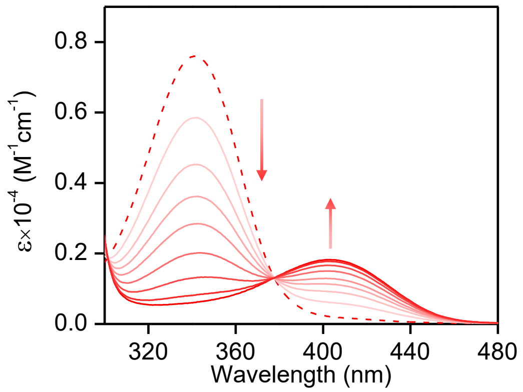

Fig. S4. UV-vis absorption spectra of PPY (10 μM) at various reaction times (2–60 min) of $AlCl_3$ (200 μM) addition in MeOH solvent at 25ºC. The increase or decreasing intensities with the reaction time are shown by arrows. The spectrum in the absence of $AlCl_3$ is depicted in broken.

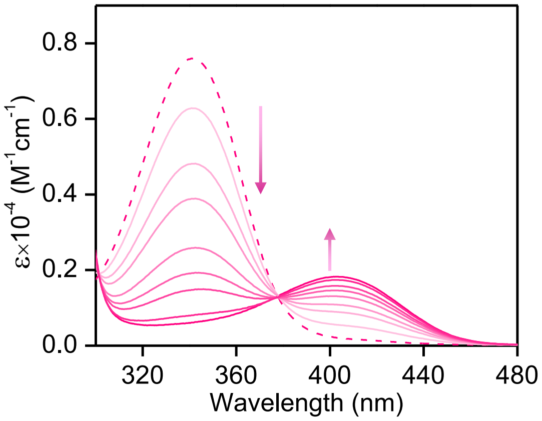

Fig. S5. UV-vis absorption spectra of PPY (10 μM) in the presence (solid) various amount of anhydrous $AlCl_3$ (20–200 μM) addition in MeOH solvent at 25ºC. The spectra were collected in 60 min of $AlCl_3$ addition. The increase or decreasing intensities with the increase of $AlCl_3$ amount are shown by arrows. The spectrum in the absence of $AlCl_3$ is depicted in broken.

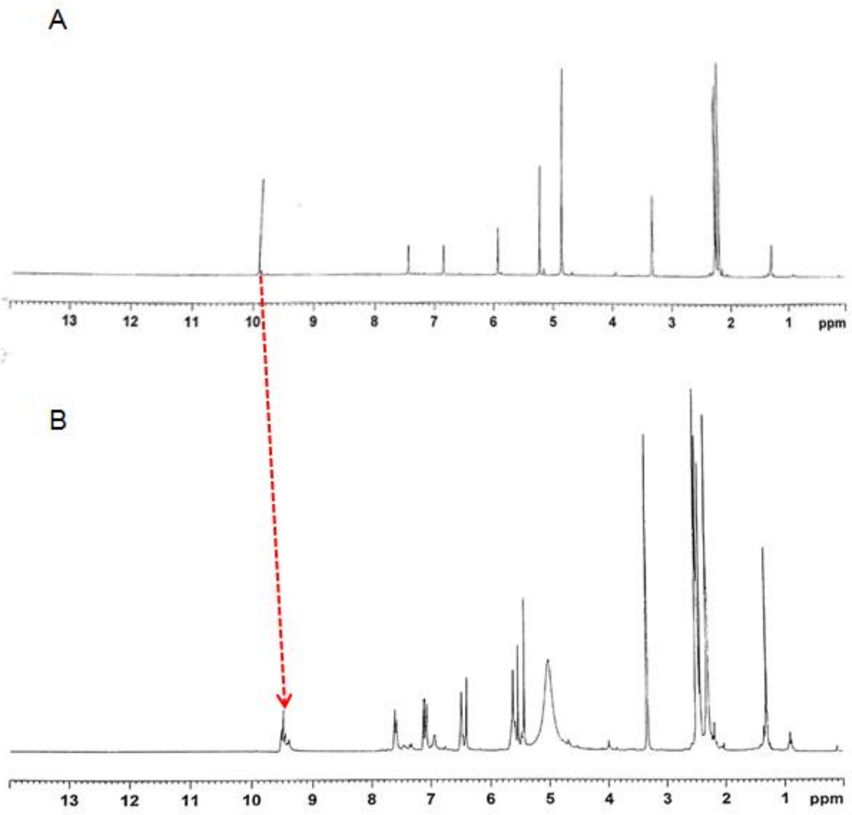

Fig. S6. ^{1}H-NMR spectrum of PPY (1 mM) in (A) the absence and (B) presence of $AlCl_3$ (20 mM) in CD_3OD.

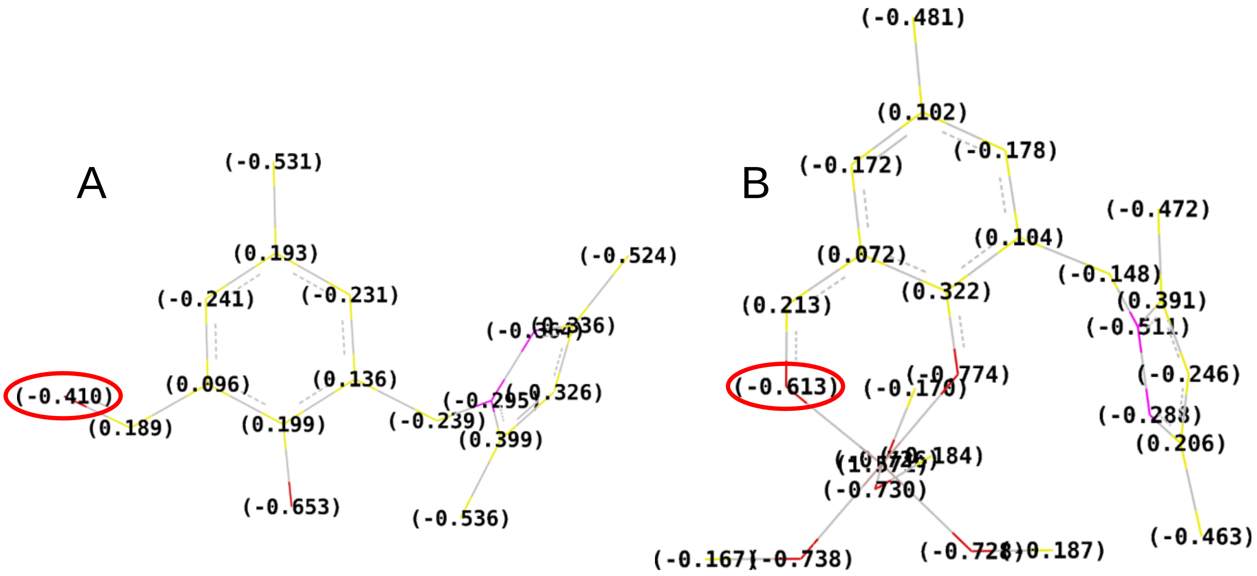

Fig. S7. Mulliken atomic charge values for the optimized geometries of (A) PPY and (B) PPY/Al^{3+} complex containing coordination of 4 MeOH molecules obtained from DFT based theoretical calculations (B3LYP/6-31G). Color index for atoms: H, cyan; C, yellow; N, pink and O, red. H-atoms are removed in optimized structure for clarity. The charge values on carbonyl-O atoms are depicted by red circles.

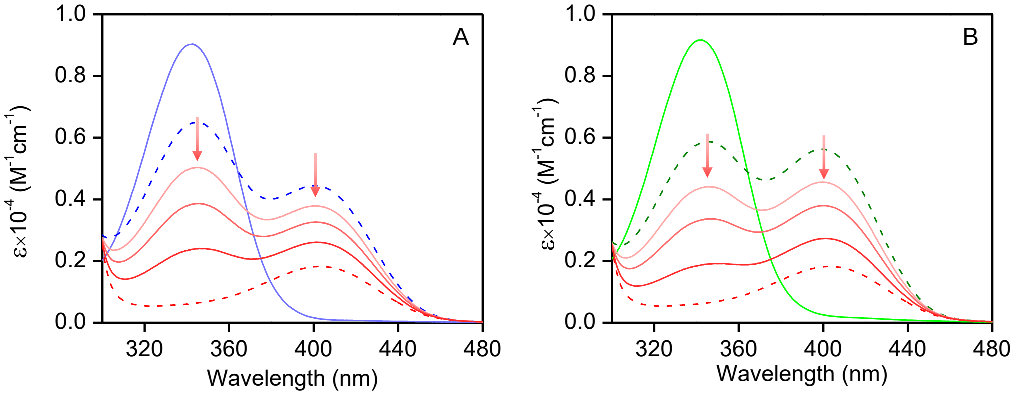

Fig. S8. UV-vis absorption spectra of PPY (10 μM) in 60 min of anhydrous $AlCl_3$ (200 μM) addition in EtOH or ^{i}PrOH (broken blue: EtOH (A), broken green: ^{i}PrOH (B)) at 25ºC. (broken red: A and B) Spectra in MeOH are shown for comparison. (solid red: A and B) Spectra recorded in 60 min (120 min of $AlCl_3$ addition) upon addition of various amount of MeOH (5–20%) in (A) EtOH and (B) ^{i}PrOH reaction mixture at 25ºC. The spectra PPY in the absence of $AlCl_3$ and MeOH are shown in light blue (A) or light green (B). The spectra were collected in 60 min of $AlCl_3$ addition. The decreasing intensities with the increase of MeOH amount are shown by arrows.

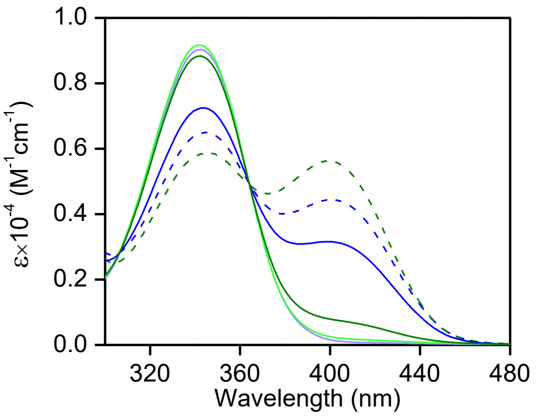

Fig. S9. UV-vis absorption spectra of PPY (10 μM) in 60 min of anhydrous $AlCl_3$ (200 μM) addition in (broken blue) EtOH and (broken green) ^{i}PrOH at 25ºC. Spectra recorded in 60 min (120 min of $AlCl_3$ addition) upon addition of 5% (*v*/*v*) water (solid blue) EtOH and (solid green) ^{i}PrOH reaction mixture at 25ºC. The spectra PPY in the absence of $AlCl_3$ and water are shown in light blue and light green (solid).

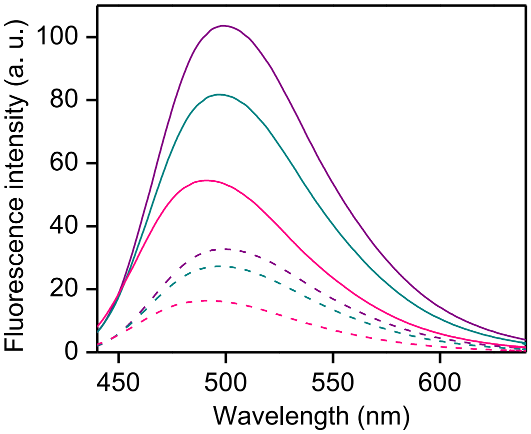

Fig. S10. Fluorescence spectra of PPY/Al^{3+} in various alcohol medium in the absence (solid) and presence (broken) of 2.5% (*v*/*v*) water at 25ºC: purple, *n*-propanol; dark cyan, ^{t}BuOH and pink, *n*-hexanol). The spectra were reordered in 60 min of $AlCl_3$ (50 μM) addition. Excitation wavelength was 405 nm.

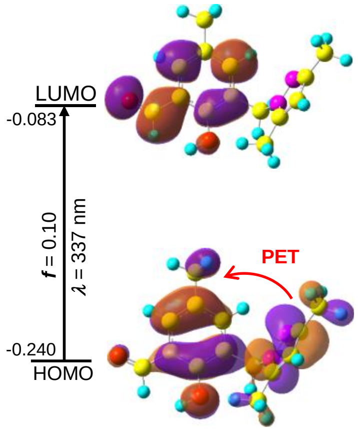

Fig. S11. Frontier molecular orbital (FMO) profile including various UV-vis absorption parameters of PPY based on TD-DFT (B3LYP/6-31G) calculations. Color index for atoms: H, cyan; C, yellow; N, pink and O, red.

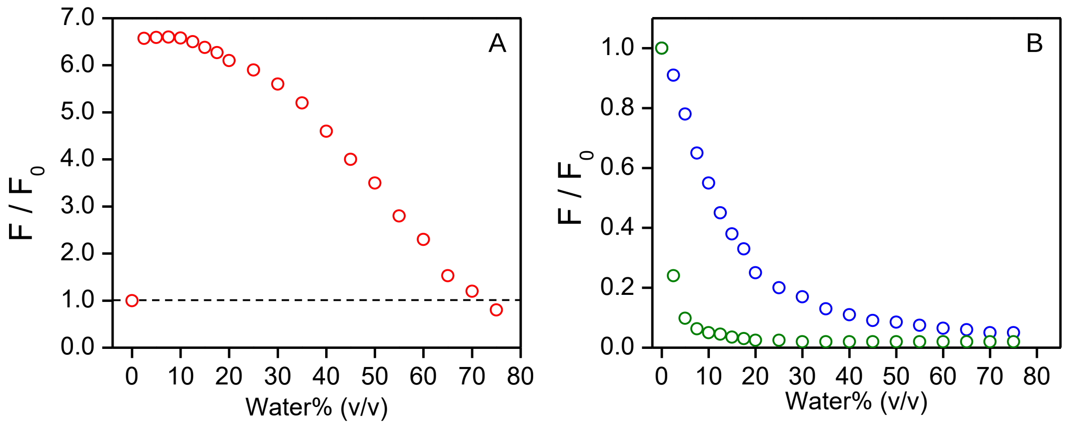

Fig. S12. The ratio of fluorescence intensities at ~505 nm for PPY/Al^{3+} in the presence of various amount of water% (*v*/*v*) and its absence are plotted with water% (*v*/*v*) in different alcohol mediums at 25ºC: (A) red, MeOH; (B) blue, EtOH and (B) green, ^{i}PrOH. The intensity values in the absence and presence of different water% are collected in 60 min of anhydrous $AlCl_3$ (50 μM) addition in the medium containing PPY (2 μM). (A) The broken black line is shown to identify the extent the increase or decrease of intensity with respect to the intensity in the absence of water. Excitation wavelength was 405 nm.

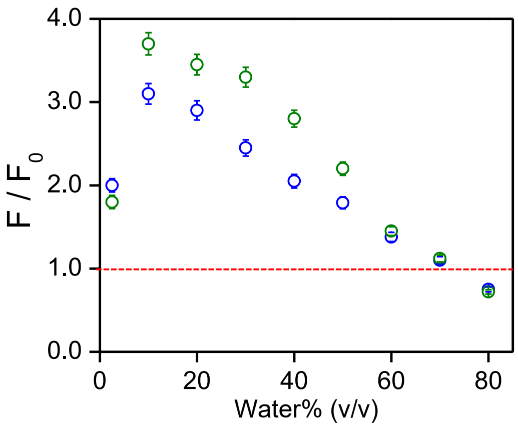

Fig. S13. The ratio of fluorescence intensities at ~505 nm for PPY/Al^{3+} in the presence of various amount of water% (*v*/*v*) and its absence are plotted with water% (*v*/*v*) in different alcohol mediums containing 10% (*v*/*v*) MeOH at 25ºC: blue, EtOH and green, ^{i}PrOH. The intensity values in the absence and presence of different water% are collected in 60 min of anhydrous $AlCl_3$ (50 μM) addition in the medium containing PPY (2 μM). The broken red line is shown to identify the extent the increase or decrease of intensity with respect to the intensity in the absence of water. The average value for each data point is obtained from triplicate measurements (n = 3).

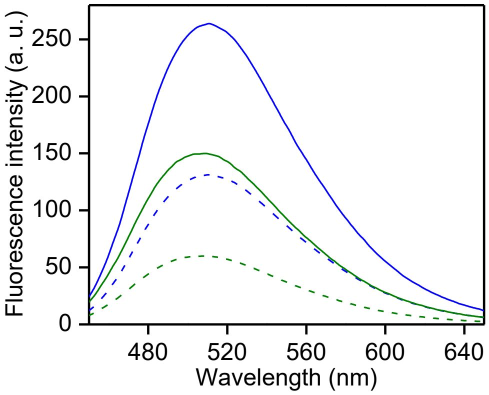

Fig. S14. Fluorescence spectra for PPY/Al^{3+} in the presence of 10% (*v*/*v*) MeOH spikes in EtOH and ^{i}PrOH medium containing 5% (*v*/*v*) water. The corresponding spectra in the presence of 10% EtOH spikes instead of 10% MeOH spike are shown in broken blue and green. Excitation wavelength was 405 nm.

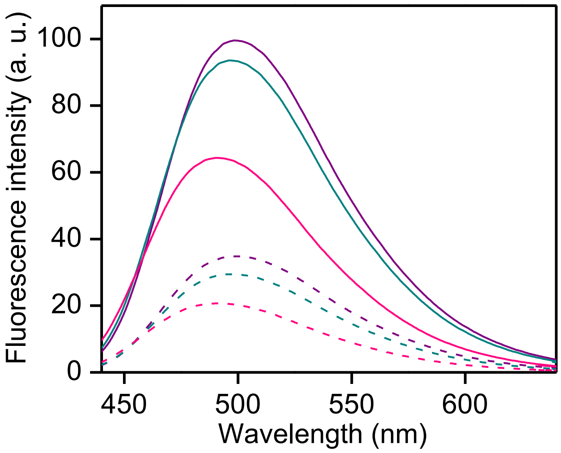

Fig. S15. Fluorescence spectra of PPY/Al^{3+} (PPY: 2 μM + $AlCl_3$: 50 μM) in various alcohol medium containing 2.5% water in the presence (solid) and absence (broken) of 10% MeOH at 25ºC: purple, *n*-propanol; dark cyan, ^{t}BuOH and pink, *n*-hexanol. The spectra were reordered in 60 min of $AlCl_3$ (50 μM) addition. The identical value of water% before and after of MeOH spike was maintained by an appropriate amount of water addition in the spiked sample. Excitation wavelength was 405 nm.

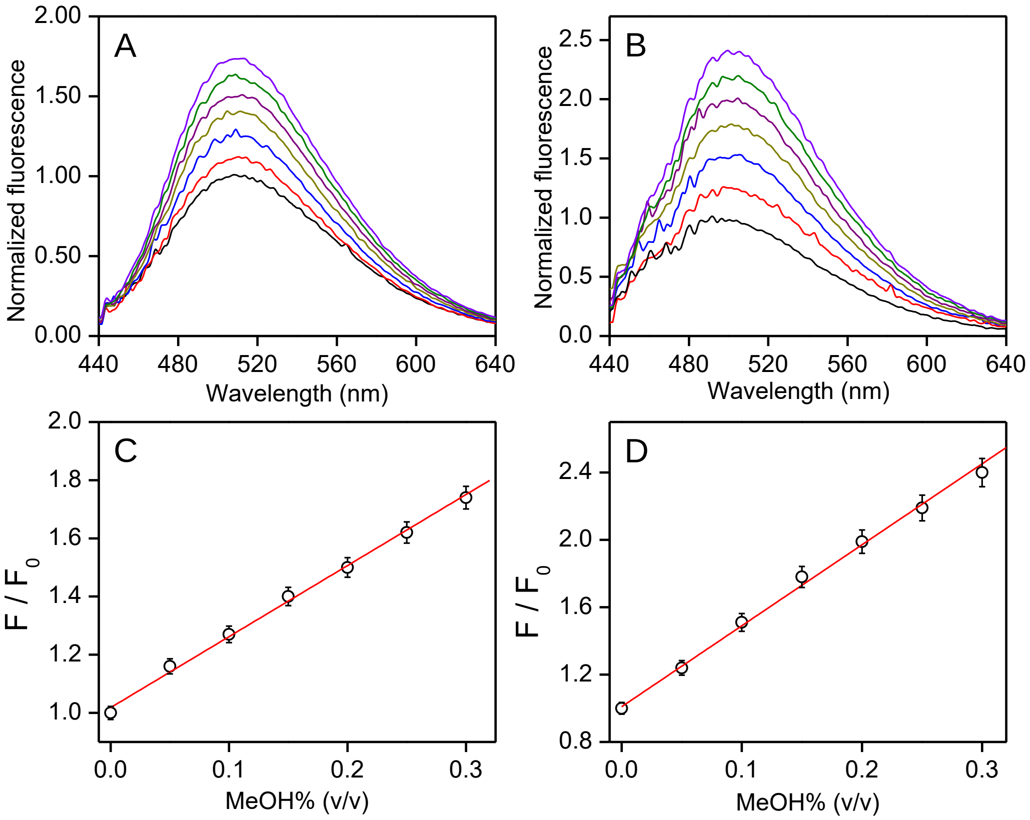

Fig. S16. (A,B) Relative fluorescence spectral changes between presence and absence of MeOH for PPY/Al^{3+} in the presence of various amount of MeOH spikes (0.05–0.30% (*v*/*v*)) in (A) EtOH and (B) ^{i}PrOH medium containing 10% (*v*/*v*) water at 25°C: red, 0.05%; blue, 0.10%; dark yellow, 0.15%; purple, 0.20%; green, 0.25% and violet, 0.30% MeOH. The spectra in the absence of MeOH spikes are shown in black. The identical value of water% before and after of MeOH spike was maintained by an appropriate amount of water addition in the spiked sample. The spectra in the absence and presence of various MeOH% are collected in 60 min of $AlCl_3$ (5 μM) addition in the medium containing PPY (0.1 μM). Excitation wavelength was 405 nm. The background fluorescence in the absence of probe was subtracted for each spectrum. (B) The maximum intensity values are plotted against the amount of MeOH spikes for (C) EtOH and (D) ^{i}PrOH. The data points are fitted by linear equation. The average value for each data point is obtained from triplicate measurements (n = 3).

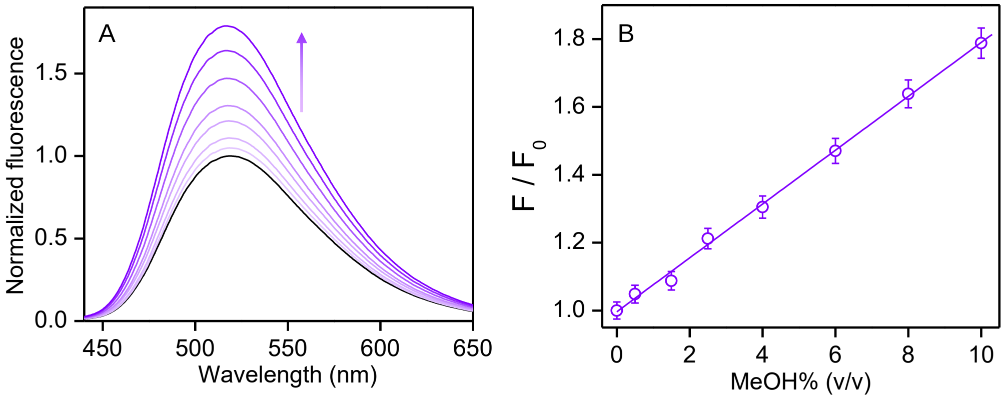

Fig. S17. (A) Relative fluorescence spectral changes between presence and absence of MeOH for PPY/Al^{3+} in the presence of various amount of MeOH spikes (0.5–10% (*v*/*v*)) in commercial wine (labelled EtOH% ~15% (*v*/*v*)) in presence of externally added 30% EtOH at 25°C. The spectra in the absence of MeOH spikes are shown in black. The identical value of water% before and after of MeOH spike was maintained by an appropriate amount of water addition in the spiked sample. The increase of intensities with the increase of spike MeOH amounts are shown by arrow. Excitation wavelength was 405 nm. (B) The maximum intensity values are plotted against the amount of MeOH spikes. The data points are fitted by linear equation. The average value for each data point is obtained from triplicate measurements (n = 3).

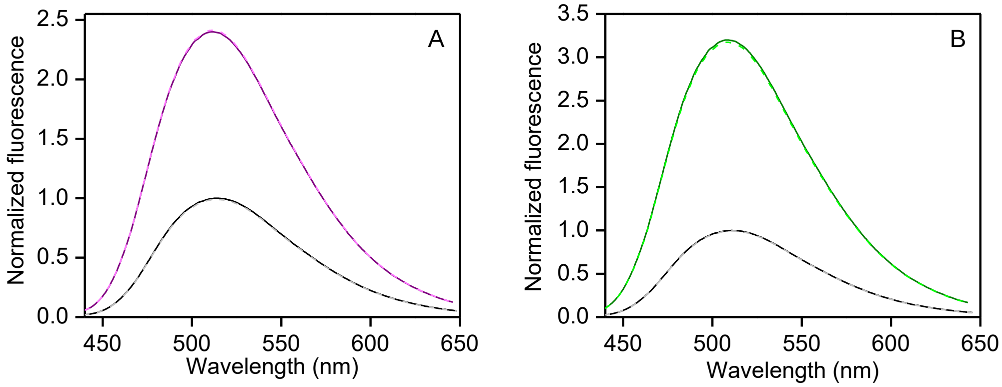

Fig. S18. Relative fluorescence spectral changes for PPY/Al^{3+} in the absence (black) and presence (A: purple and B: green) of 10% MeOH spikes in (A) EtOH/water (water: 80% (*v*/*v*)) and (B) ^{i}PrOH (water: 75% (*v*/*v*)) medium containing 1.45% (*v*/*v*) glycerol and 0.125% (*v*/*v*) H_2O_2. The corresponding spectra in the absence of glycerol and H_2O_2 are shown in broken light colors. The identical value of water% before and after of MeOH spike was maintained by an appropriate amount of water addition in the spiked sample. Excitation wavelength was 405 nm.

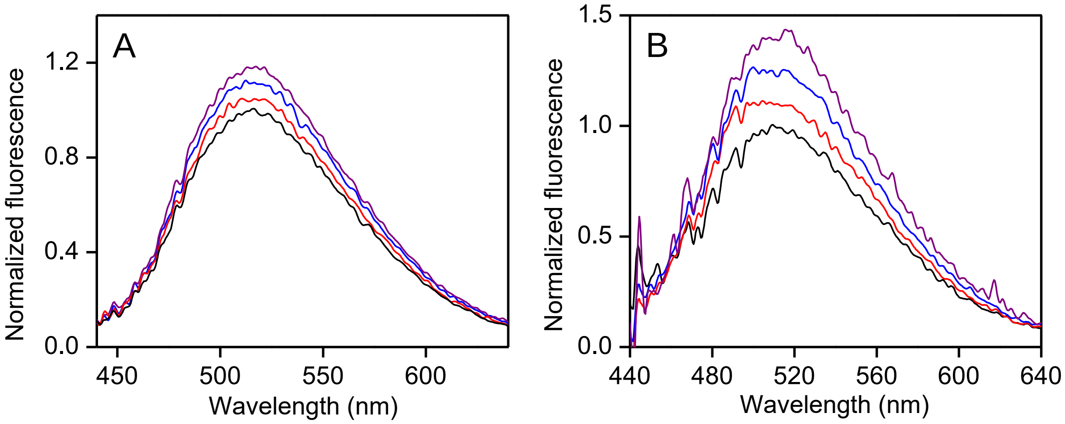

Fig. S19. (A,B) Relative fluorescence spectral changes between presence and absence of MeOH for PPY/Al^{3+} in the presence of various amount of MeOH spikes (0.03–0.18% (*v*/*v*)) in (A) commercial vodka (labelled EtOH% ~45% (*v*/*v*)) and (B) ^{i}PrOH-based hand sanitizers (labelled ^{i}PrOH ~75%) at 25°C: (A): red, 0.06%; blue, 0.12%; purple and 0.18% MeOH for vodka and (B): red, 0.03%; blue, 0.06%; purple and 0.10% MeOH for hand sanitizer. The spectra in the absence of MeOH spikes are shown in black. The identical value of water% before and after of MeOH spike was maintained by an appropriate amount of water addition in the spiked sample. The spectra in the absence and presence of various MeOH% are collected in 60 min of $AlCl_3$ (5 μM) addition in the medium containing PPY (0.1 μM). The background fluorescence in the absence of probe was subtracted for each spectrum. Excitation wavelength was 405 nm.

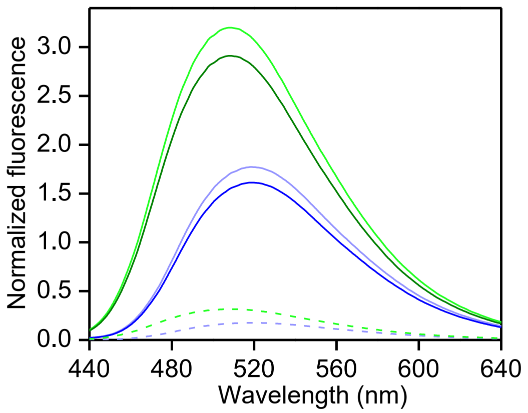

Fig. S20. Fluorescence spectra for PPY/Al^{3+} (PPY: 2 μM + $AlCl_3$: 50 μM) in (broken light blue) vodka or (broken light green) ^{i}PrOH-based sanitizer containing 10% MeOH and 50 μM EDTA. The corresponding spectra in the absence of EDTA are shown by solid light colors. Furthermore 100 μM of $AlCl_3$ was added in the solutions containing EDTA, and the spectra were recorded in 60 min of the $AlCl_3$ addition (solid blue for votka and solid green for ^{i}PrOH-base sanitizer). Excitation wavelength was 405 nm.
